# Evaluation of animal models of neurobehavioral disorders

**DOI:** 10.1186/1744-9081-5-11

**Published:** 2009-02-25

**Authors:** F Josef van der Staay, Saskia S Arndt, Rebecca E Nordquist

**Affiliations:** 1 Program 'Emotion and Cognition', Department of Farm Animal Health, Veterinary Faculty, Utrecht University, PO Box 80166, 3508 TD Utrecht, the Netherlands; 2 Division of Laboratory Animal Science, Department of Animals, Science and Society, Veterinary Faculty, Utrecht University, the Netherlands

## Abstract

Animal models play a central role in all areas of biomedical research. The *process *of animal model building, development and evaluation has rarely been addressed systematically, despite the long history of using animal models in the investigation of neuropsychiatric disorders and behavioral dysfunctions. An iterative, multi-stage trajectory for developing animal models and assessing their quality is proposed. The process starts with defining the purpose(s) of the model, preferentially based on hypotheses about brain-behavior relationships. Then, the model is developed and tested. The evaluation of the model takes scientific and ethical criteria into consideration.

Model development requires a multidisciplinary approach. Preclinical and clinical experts should establish a set of scientific criteria, which a model must meet. The scientific evaluation consists of assessing the replicability/reliability, predictive, construct and external validity/generalizability, and relevance of the model. We emphasize the role of (systematic and extended) replications in the course of the validation process. One may apply a multiple-tiered 'replication battery' to estimate the reliability/replicability, validity, and generalizability of result.

Compromised welfare is inherent in many deficiency models in animals. Unfortunately, 'animal welfare' is a vaguely defined concept, making it difficult to establish exact evaluation criteria. Weighing the animal's welfare and considerations as to whether action is indicated to reduce the discomfort must accompany the scientific evaluation at any stage of the model building and evaluation process. Animal model building should be discontinued if the model does not meet the preset scientific criteria, or when animal welfare is severely compromised. The application of the evaluation procedure is exemplified using the rat with neonatal hippocampal lesion as a proposed model of schizophrenia.

In a manner congruent to that for improving animal models, guided by the procedure expounded upon in this paper, the developmental and evaluation procedure itself may be improved by careful definition of the purpose(s) of a model and by defining better evaluation criteria, based on the proposed use of the model.

## Background

Animal models play a central role in the scientific investigation of behavior and of the (patho)physiological mechanisms and processes that are involved in the control of normal and abnormal behavior [1-7]. When talking about animal models we almost always implicitly assume that they are meant to model humans (or a species other than the one investigated; [8]); i.e. that they focus on the homology/analogy of the behavior and underlying substrate in the model animal with that in humans. Despite the long history of using animal models in the investigation of neuropsychiatric disorders (see [9]) and the central role they play in biomedical research in general, the *process *of model building, development and evaluation has rarely been addressed systematically.

We define animal models in the behavioral neurosciences, which include models of neurobehavioral disorders, as follows:

*An animal model with biological and/or clinical relevance in the behavioral neurosciences is a living organism used to study brain-behavior relations under controlled conditions, with the final goal to gain insight into, and to enable predictions about, these relations in humans and/or a species other than the one studied, or in the same species under conditions different from those under which the study was performed *[10].

The model of a neurobehavioral disorder must be broken down into elemental phenotypes that are observables (i.e. elements that can be observed and measured directly), measurables (i.e. elements that can be assigned a qualitative or quantitative attribute) and testables (i.e. are measurables that can be submitted to statistical evaluation in order to test and confirm – or falsify – a hypothesis) [11], which should preferentially be testable in both humans and animals [12,13]. These testables need to be defined operationally.

Recently, there is a strong focus on defining endophenotypes, i.e. characteristics related to the phenotype of primary interest. Endophenotypes must first be detected and validated using the data of patients and their (first degree) relatives [14], although the search may be guided and their validation supported by animal studies [15]. Then, attempts can be made to translate these endophenotypes to animal models. Endophenotypes may be behavioral, i.e. cognitive, neuropsychological, (psycho)physiological [16], biochemical, endocrinological, or neuroanatomical [17,18]. Endophenotypes are hypothesized to mediate the impact of gene products on the phenotype under study, i.e. they are considered as symptoms (phenotypes) with a clear genetic connection. Endophenoptypes may in some sense be "closer to the genes" than the key symptom as described according to the psychiatric nosology [18,19]. Identifying endophenotypes and basing models on endophenotypes may facilitate generalization of results from the model species to other species, including humans.

In this paper we'll focus in particular on the model evaluation stage that is part of an iterative process involved in developing animal models [10]. The starting point of the process of model building is the definition of the purpose(s) of the model [10,20]. Then, the model is developed and tested. The evaluation of the model takes into consideration the questions it is expected to answer, its validity – in particular predictive, construct [21] – and external validity or generalizability [22]. Simultaneously, it takes animal welfare issues into account [23,24].

We will first review the purpose of animal models, and will then introduce and explain the concepts reliability, replicability, different forms of validity, and the concept of animal welfare. They are all relevant in the model evaluation process, where they serve as evaluation criteria. Next, we propose a workflow for model building and model evaluation. The role of replications in this process will be highlighted. Finally, we perform a model evaluation of the neonatal hippocampal lesions as a model of schizophrenia, that is guided by the described workflow and we address some recent concerns about the translation of the results obtained in "standard" animal models to humans. We suggest that systematic model building and evaluation and the application of strict evaluation criteria improves the translational properties of animal models.

### Purpose of animal models

Animal models are developed and used for varying purposes [1,25,26]. Explicit statements about the (expected) purposes of a model are necessary to define criteria for model building, model evaluation and model use [1,20,27,28]. The explicit definition and designation of the specific purposes that an animal model should fulfill is basic as it allows to define a set of weighted criteria for evaluating the model [26]. These criteria, combined with criteria of reliability, replicability and validity are used in the model evaluation stage. Of course it will not always be possible to anticipate whether the model will accomplish the intended purpose. Thus, one starts with assumptions which should be tested in a continuous process. If evidence accumulates that the intended goal/purpose cannot be reached, then one should consider abandoning further development of the model [29].

The purpose already determines the generality of the answers a model can provide [26]. It is, for example, of importance to define whether the 'full blown pathology', the syndrome, or 'specific aspect(s)' of neurobehavioral disorder, e.g. specific symptoms, are to be modeled [1,27]. However, trying to model the entire pathology is seen as an unrealistic attempt (e.g. [30,31]). Simply abandoning the term "model" because it is highly unlikely that it can mimic the full blown pathology or syndrome, as suggested by O'Neil and Moore [31], will not help to improve animal experimentation. Unfortunately, we often do not yet understand the full pathophysiology of a disease and are therefore compelled to focus on specific aspects of the neurobiological disorder [32]. In the long run, attention should shift from modeling the symptomatology of a disease to unraveling the pathological mechanisms behind a disease [6]. However, the best achievable quality of an animal model of a neurobehavioral disease is delimited by the state of knowledge about the disease.

An overview of different types of 'model animals' (part A), the independent and dependent variables in animal models (part B), and the sources of criteria for developing and evaluating the model (part C) is provided in Table 1[33,34]. An extended discussion of the different animal models can be found in [10].

**Table 1:** Overview of animal models.

**A: Types of 'model animals'**		
**Normal animals**	**Animals with naturally occurring deficits**	**Animals with experimentally induced deficits**

Normal subjects, i.e. animals without any observable behavioral deficit Note: At first glance, normal animals are not model animals for the study of neurobehavioral disorders. However, they may be used for drug screening, or as in vivo (behavioral) bioassay. Normal animals are used to • assess the safety/toxicology risk of a putative therapeutic; • obtain an estimate of the putative abuse liability of a compound; • explore the neurobiological specificity of compounds and their molecular and cellular mechanisms of action In addition: studying behavior in normal animals provides the baseline data for identifying abnormal behavior	Spontaneously and endogenously occurring psychiatric or neurological conditions; spontaneously occurring mutations; aging animals Genetic lines: inbred strains and their crossings Selection lines, established through artificial selection favoring low and/or high values of a particular trait within a given population for a number a successive generations; preferentially including an unselected control line for comparison Selected extremes from a particular animal population, e.g. good vs. poor learners, dominant vs. subordinate animals, non-aggressive vs. aggressive animals	Transgenic and knockout animals; Chromosomal substitution strains; animals from mutagenesis screens (after thorough phenotyping and validation) Selection lines resulting from selective breeding Environmental factors: e.g. animals experiencing acute or chronic stress, pain, or sleep deprivation Animals with disruptions induced by dietary composition (e.g. tryptophan depletion), pharmacological compounds (e.g. scopolamine, MK-801), or electrically, or by hypoxia or anoxia Animals with focal or global ischemic, embolic, or hemorrhagic cerebral stroke Animals with CNS-specific lesions: neuro- or immunotoxic lesions; lesions induced by aspiration, ablation (knife cuts); radio-frequency lesions, cryogenic lesions

**B: Independent and dependent variables**		

**Independent variable (the model animals listed in part A)**	**Dependent variables and/or (endo)phenotypes**	
	
	**Neuropathological changes**	**Behavioral changes**

E.g. genetically modified animal, aged animal, lesioned animal, ischemic animal, hypoxic animal, aged and lesioned animal, i.e. combination of deficits (see part A)	Damage or dysfunctions induced: site and size of neuronal damage (neuropathology), effects on specific neuronal circuits or neurotransmitter systems, psychophysiological and biological (endo)phenotypes	Behavioral dysfunction or malfunction: impaired cognitive performance, impaired sensorimotor functions, neuropsychiatric symptoms, behavioral (endo)phenotypes
	Homology of damaged area(s) or neuropathological changes.	Homology of disrupted processes or impaired functions
	Operational definition(s) of the neuropathological (endo)phenotype(s)	Operational definition(s) of the behavioral (endo)phenotype(s)

**C: Setting criteria for model building and model evaluation**		

	Experts: clinicians, pathologists, molecular biologists, etc., depending on which aspects of the animal model are considered	Experts: behavioral scientists such as psychiatrist, (bio)psychologists, ethologists, behavioral pharmacologists
	Experts must: • define as exactly as possible the (neuro)pathological symptoms to be modeled; • elaborate a set of model evaluation criteria	Experts must: • define the behavioral (dys)functions to be modeled as precisely as possible, possibly derived from clinical diagnostic criteria; • elaborate a set of model evaluation criteria, which may be derived from psychological test theory for the tests applied to assess the dysfunctions

Part A lists different types of 'model animals' for the study of behavioral dysfunctions (modified from [33]). It focuses on the type of subject (independent variable) and is not concerned with the type of dependent variable measured

In part B, (modified from [10]), the independent and the dependent variables in deficiency models are tabulated.

In part C, the expertise needed to define criteria for building a 'good' animal model and for its evaluation is listed. The validity of a model should be determined using multidisciplinary, interdisciplinary, or – preferentially – transdisciplinary approaches (the latter addressing a common problem against the background of a shared conceptual framework by employing theories, concepts and scientific methods of the different disciplines involved; [34]). No explicit set of rules exists for the mental, physical, and/or neuro(patho)logical changes (2^nd ^column) that are considered to cause the behavioral dysfunctions (3^rd ^column).

The purposes of animal models of neurobehavioral disorders usually are:

● first, to enhance our understanding of the underlying substrates and mechanisms controlling normal and abnormal behavior, i.e. the brain-behavior relation. This is done experimentally by, for example, inducing dissociations between processes, sub processes and modulating influences, either pharmacologically, through the destruction of neural tissue, or by using animals with naturally occurring deficits [35]. Investigating the naturally occurring or experimentally induced brain damage and its consequences should help to elucidate the primary and secondary sequelae and unravel their underlying deleterious molecular cascade [36] (see Table 1, part A).

● second, to translate these insights from the preclinical animal study to the clinic (and vice versa [37]), through

▶ identifying new targets, pathways and mechanisms of drug action [38-40];

▶ assessing the effects of putative neuroprotective, anti-degenerative, revalidation-supporting, mental health promoting, and/or cognition-enhancing compounds or treatments [27,41-44], and assessing risks (safety, teratology, toxicology) associated with these treatments [45].

### Validity of animal models

Validation of a model is a scientific method to improve the confidence in a model, i.e. to evaluate its plausibility and consistency. Validity is defined as *„(..) the agreement between a test score or measure and the quality it is believed to measure." *[46], p. 131). It is not a demonstration of the "truth" of a model. One validates, not an animal model, but the interpretation of the data arising from this model. Validity in that sense is a major criterion for evaluating animal models [1]. No animal model can be valid in all situations, for all purposes. Validity is restricted to a specific use of the model, and consequently, must always be open for discussion and re-evaluation [47].

There is no general consent about how to weigh the different categories of validity in the model evaluation process. We hold that the validation process should consider the reliability and replicability (internal validity), predictive validity, construct validity, and external validity (i.e. generalizability) of a model. The concepts of internal validity, face, predictive and construct validity have been elucidated in a number of publications (e.g. [10,48,49]). Here, we provide a short description of these concepts.

#### Reliability and replicability, internal validity

Reliability is primarily a quality of the assessment instrument, whereas replicability or reproducibility is a quality of the results obtained using a particular animal model. Reliability thus indicates how consistent an assessment/testing device/method is, i.e. it expresses the extent to which a measurement instrument yields consistent results each time that the measurements are performed under the same experimental conditions. Replicability or reproducibility is the degree of accordance between the results of the same experiment performed independently in the same or different laboratories [50,51].

Internal validity refers to the quality of the experimental evaluation of the animal model, i.e. to how well a study was performed, how strictly putative confounding variables were controlled, and how confident one can be that the changes observed in the dependent variable(s) are *caused *by experimentally manipulating the independent variable(s), and not by confounds, i.e. factors that might also affect the independent variable and may offer alternative explanations of the results obtained [22]. It does not make sense to speculate about the external validity/generalizability of experimental studies outside the laboratory, in the 'Outside World' or "Real World", as long as it has not been verified that the results are valid within the laboratory (internal validity; [22,52]). High reliability and replicability are the foundation of good internal validity.

#### Face validity

Face validity is the degree of descriptive similarity between, for example, the behavioral dysfunction seen in an animal model and in the human affected by a particular neurobehavioral disorder. Similarity of symptoms in fact may be the starting point of indentifying a potential animal model of neurobehavioral disorders [53]. Although face validity has been proposed to constitute a major (or even the most important; e.g., [1]) criterion for model evaluation, the strong emphasis on this criterion has been criticized (e.g., [21]). Natural selection may operate on the consequences of behavior, not on the behavior *per se*, and therefore, the consequences of the behavioral pattern, not the behavior itself, may be isomorphic [54]. Moreover, it is conceivable that species-dependently, similar behaviors could serve different functions or that different behaviors serve the same function. Consequently, the same behavioral dysfunctions may be the expression of different underlying physiological or psychological states [3]. Demanding face validity may thus prove to be an unrealistic criterion [2]. It incorporates the risk of anthropomorphic reasoning, which may retard or even prevent the development of relevant animal models [1]. Too strong an emphasis on face validity may also form an obstacle for developing animal models using phylogenetically lower animal species [55], as the similarity of symptoms [49] is generally higher in species that are phylogenetically closer to humans (see comments by [56]). In agreement with Sarter and Bruno [21], we consider face validity as a criterion of less importance in appraising an animal model of neurobehavioral disorders. A lack of face validity does not *per se *invalidate a model [3,10]. In any case, animal models with face validity have to go through the scientific process of establishing their predictive and construct validity [53].

#### Predictive validity

An animal model with high predictive validity (also called criterion validity; [57]) predicts behavior in the situation it is supposed to model, i.e. it allows extrapolation of the effect of a particular experimental manipulation from one species to other species, including humans, and from one condition (e.g. the laboratory) to the other (e.g. the 'Real World'), or from one testing timepoint to another [57]. Predictive validity may share components of generalizability (or external validity; see below) of a model.

A narrower concept of predictive validity is used in psychopharmacology (e.g. [58-62]) where it is considered to be of particular importance in drug development programs [63]. In this context, predictive validity refers to the ability of a drug screening or an animal model to correctly identify the efficacy of a putative therapeutic [64]. However, in diseases with a poor therapeutic standard, only a few weakly effective compounds may be available in the clinic, which hardly can be used to determine the predictive validity of animal models. A consequence of relying too heavily on the predictive validity as most crucial criterion is that these animal models may be unsuited to detect novel therapeutic principles [21].

#### Construct validity

According to Epstein [57] construct validity points to the degree of similarity between the mechanisms underlying behavior in the model and that underlying the behavior in the condition, which is being modeled. Construct validity thus is a theory-driven, experimental substantiation of the behavioral, pathophysiological, and/or neuronal components of the model [21], i.e. it reflects the degree of fitting the theoretical rationale and of modeling the true nature of the symptoms/syndrome to be mimicked by the animal model [1]. Constructs define a framework of theoretically relevant relations [46,47] that reflects the soundness of the theoretical rationale [64]. Construct validity expresses the goodness of fit between the relationship of the manipulations (i.e. independent variables) and of the measurements (dependent variables) with the theoretical hypotheses to be tested [65]. In agreement with Sarter and Bruno [21], we argue that construct validity is the most important criterion for animal models because it addresses the soundness of the theory underlying the model, and because it provides the framework for interpreting data generated by the model.

#### External validity/Generalizability

Assessment of the generalizability (or "external validity") of experimental findings should be integral part of the model building process. *External validity *is the extent to which the results obtained using a particular animal model can be generalized/applied to and across populations (and eventually, species) [66] and environments, or "the extent to which experimental findings make us better able to predict real-world behavior" [67]. The assessment of the external validity is an empirical process. This process may be performed by *systematic replications *or *differentiated replications*, i.e. replications of the original studies in which a particular set of independent variables is varied systematically in order to evaluate whether the results obtained are robust across, for example, rearing and housing conditions, ages, gender, and test conditions or tests used. Ideally, a replication study is not a mere repetition of an earlier study, but should extend the scope of previously performed studies, allowing statements about the generality of results [68].

It is generally accepted that the measures usually taken to increase internal validity may compromise external validity/generalizability [51,69,70], simply because they restrict the range of conditions under which the relationship between dependent and independent variables is being tested. On the other hand, higher internal validity fosters higher explanatory power [71].

Whatever classification system is being used, determination of the generalizability/external validity should constitute a key feature of the model building process. A number of factors, such as rearing and housing environments, gender and age of the animal, and the exact testing conditions, have an impact on the generalizability of findings originating from an animal model.

### Replications in model building and model validation

Replicability of results is fundamental in empirical research and is one of the pillars of science [72-74]. Experimental results are preliminary as long as they have not been corroborated, preferably by investigators other than those who originally performed the investigations [10,75]. Replications are essential for determining the reliability/replicability, and external validity/generalizability of a model.

Often, the original study will suffer from poor statistical power due to the small number of animals involved. Reasons for underpowered studies may be the restricted availability of model animals, or the drive and objective of ethical committees and regulatory authorities to minimize the number of animals permitted in a study [76]. In that case, successful replications will increase the confidence in the results and implications of the study [77].

**Figure 1:**
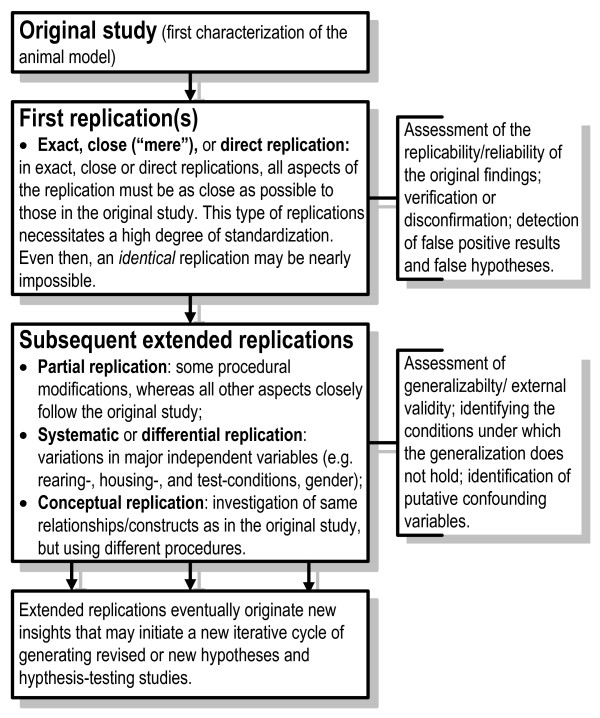
**Replication studies in the model validation process**. Replication studies can be used in a two-tiered approach to assess the reliability/replicability and generalizability/external validity of experimental findings.

**Figure 2:**
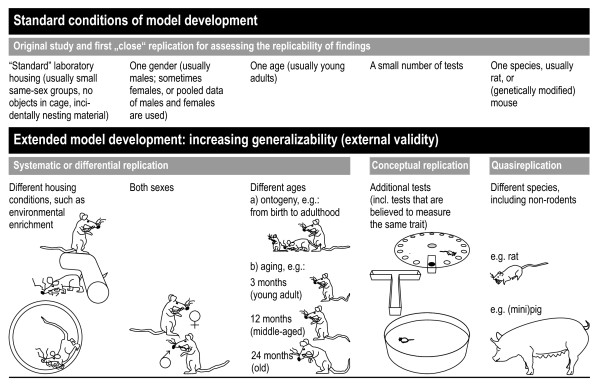
**Increasing the generalizability (or external validity) of a model**. This can be achieved by assessing the effects of rearing and housing conditions (first column) through partial, systematic, and conceptual replications (see Fig. 1). Gender effects (second column), ontogenetic and aging effects (third column) should be an integral part of the model building process. In addition, the battery of tests for assessing the dependent variables (see Table 1, Part B, second and third column) should be extended and should include tests that are believed to measures the same trait/construct (fourth column; e.g. the Barnes maze [78], the T-maze [80], and the Morris maze [79] may be used to assess spatial working memory performance). Quasireplications are not part of the model building process, but may be used for assessing the generalizability across species.

One may apply a "replication battery" to estimate the reliability/replicability (internal validity) and generalizability (external validity) of the results of the first, original study. This replication battery can be conceived as a two-, or if warranted multiple-tiered, experimental approach [72] (see Figs. 1 and 2; [78-80]).

The first step consists of determining the *replicability/reliability*, i.e. the *internal validity *of the original findings. To this end, the replication study should as close as possible, with high precision and accuracy, repeat the original study [74]. These studies are called "close" [68], "exact" [72], or 'direct' [73] replications. Standardization, including the specific strain/subline used [81] is a sound basis for assessing the replicability/reliability of results. Even the most accurate repetition of a study, however, will deviate from the previous one to some degree, i.e. a close replication will already provide first estimates of the generalizability of a study. If a replication study fails to corroborate the results of the original study, either the original study or the replication study may reflect false findings [73,74].

The second step consists of determining the *generalizability *or *external validity *of results. This is achieved through extending the replication by varying the levels of relevant factors in the repetition (called: "systematic replication" [73] or "differentiated replication" [68]). In these replication approaches, major aspects of the experimental conditions are (systematically) varied, such as rearing and housing environments, gender and age of the animal, and the exact testing conditions that may have an impact on the generalizability of findings originating from an animal model (see above). In "partial replication" studies (slight) procedural modifications are introduced whereas all other aspects closely mimic the original study (a "true" replication according to [72]). Conceptual replications investigate the same relationships/constructs as the original study, using different procedures (a "true" replication according to [72]). In quasireplications, species different from the one used in the original study are tested ([77], see Fig 2, last column). Quasireplications are a first step to develop an animal model in a different species and may initiate a new process of model building and model evaluation.

**Figure 3:**
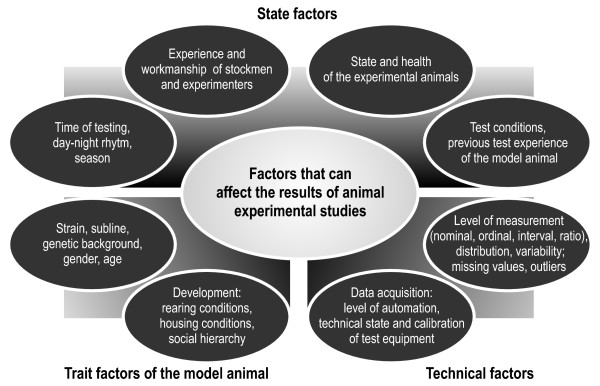
**Factors affecting the results of animal experimental studies**. In order to increase internal validity, care must be taken to identify, control and/or eliminate confounding factors (after [83]).

Some of the major factors that should be taken into account for replications are the effects of the *rearing and housing conditions *(e.g. [82-84]), *gender differences *[85,86], and the *age of the animals*, such as ontogenetic aspects, [87-91], and the effects of aging, [92,93] (see Fig. 2). Factors that might affect the behavioral phenotype of an animal model may in principle be investigated systematically during the model validation process. A number of these factors are depicted in Fig. 3[83]. Whereas the abovementioned factors (e.g. environment, gender, aged) can be varied systematically in controlled experiments, others are laboratory specific. These factors act as confounds and are held responsible for poor replicability of results across laboratories. (e.g. [84,94]). To complicate matters further, these factors might interact in multiple ways [83].

*Multiple behavioral tests *(see Fig. 2, fourth column) should be applied that approximate the range of symptoms characterizing the disease/symptomatology to be modeled (e.g. [95-97]), including different tests with different end-points that are believed to tap the same underlying states and traits [20,61,98-100]. Eventually, to assess the generalizability of a model, the tests should be applied under a range of testing conditions such as, for example, dietary regimes [101], or behavior-modulating drugs [92], which may challenge the system. The effects of these experimental manipulations should be investigated in a later stage of the process of model building and development.

It has been questioned whether each successful replication must reject the null-hypothesis (H_0_: no effects of the experimental manipulations) [72], and whether the failure to replicate may reflect a type II error [102]. In any case, the direction and size of effects should be replicable [68].

Extended replications allow identifying the conditions under which the generalization does not hold, and they contribute to detecting putative confounding variables and assessing their effects [68]. These replications expose the strength and weaknesses of findings and the limits of their generality. Extended replications eventually generate new insights that may initiate a new iterative cycle of generating revised or new hypotheses and in its wake hypothesis-testing studies [103].

Standardization of the breeding, housing and sampling/testing conditions is crucial for ensuring consistency among investigators and comparability of data across different laboratories [81,83,104-106] and over time [94]. They are needed to build up appropriate databases integrating results from different laboratories with the aim to characterize the phenotypes of the model animal (e.g. [83,105,107,108]), and for establishing databases with normative data of background and reference strains [109,110]. Standardization of test conditions is also crucial for test validation.

While it is possible that housing and testing animals under standardized conditions may yield singular or "idiosyncratic" findings (e.g. [70,111]), this will be detected as soon as one tries to replicate the study, or modifies housing or testing conditions (see above) as part of the refinement of the model or of determining the external validity of a model. Some anticipate that strict standardization may reduce the odds of serendipitous or unexpected findings, in particular due to a diminished diversity in the experimental approaches [112] and because too rigid standardization bears the risk of overlooking or missing interesting phenomena [113]. These putative disadvantages, however, don't outweigh the scientific benefits of standardization.

Summarizing, face validity is at the *naive *level, i.e. the test *looks like *it is valid, because of the perceived resemblance (isomorphy) between the model and the situation or process to be modeled [64]. Predictive validity is at the *empirical *level, i.e. data show that the outcome obtained in the model has some predictive value for the situation or process to be modeled. Construct validity is at the *theoretical *level. Finally, generalizability/external validity is at the *empirical *level, and comprises components of both predictive and construct validity. One can also say that face validity reflects the isomorphic aspect, predictive validity the correlational aspect, construct validity the homologous aspect [114,115], and generalizability/external validity the relevance of a model (i.e. the ability to make scientifically sound and relevant predictions about the "Real World").

### Animal welfare and minimized discomfort: ethical criteria for model evaluation

Most students of (ab)normal behavior and neurobehavioral disorders will adhere to a utilitarian view on the use of model animals [116-118], i.e. animal experimentation is justified by the expected benefits for humans (and eventually other animals) [118]. This does not rule out the obligation to take into consideration animal welfare, and to take any action needed to reduce discomfort and pain [119]. Only very few publications address welfare considerations in the context of animal model building and evaluation (e.g. [120]). Animal welfare should be matter of course in animal research [30,118] and should be an integral part of evaluating animal models [23,121].

#### The five freedoms

A complicating factor in safeguarding animal welfare is that the concept itself is only poorly defined and consequently, difficult to translate into measurables [24,69,122,123]. It is predominantly based on the principle of the 'five freedoms', i.e. 1) freedom from thirst, hunger, and malnutrition; 2) suffering; 3) pain, injury, and disease; 4) freedom to express normal behaviour, by providing sufficient space, proper facilities and company of the animal's own kind; and 5) freedom from fear and distress [124]. The underlying idea is that animals should be reared, housed, and tested under conditions that allow maintaining or restoring homeostasis.

● Unfortunately, measuring pain and its emotional components in animals objectively is an underdeveloped field of research. Scientists so far mainly depend on the pure assumption that due to our evolutionary relatedness, everything that is perceived as painful in humans potentially also causes pain in animals (see also [69]).

● Stress is adaptive in nature but can in parallel comprise negative consequences for health and welfare [125,126]. There is, however, no simple physiological or behavioral criterion that marks the point at which stress turns into distress [127]. Thus, one could argue that it is the ethical obligation of science to develop methods which allow for the objective measurement of (di)stress levels.

● Sensorimotor impairments and disabilities might negatively influence the welfare of animals being used. Consequently, the application of tests for sensorimotor functioning like those described in the Irwin [128] or SHIRPA [93,129] protocols should be an integral part of model development and evaluation. If sensorimotor dysfunctions are detected it is common practice to select test systems that are not dependent upon the compromised sensory and/or motor function, at the risk of neglecting possible discomfort of the animals. Although discomfort may inevitably be part of animal models of neurobehavioral disorders [130,131], a careful evaluation must enable us to decide whether the observed dysfunctions and associated discomfort are part of the phenotype under consideration, or whether action is indicated to reduce the discomfort.

● Anxiety is a biologically relevant adaptive behavioural response and therefore not negative by nature. A clear distinction has to be made between "normal" and inappropriate anxiety-related responses. It is self-evident that scientists must avoid procedures causing *unnecessary *anxiety in animals. The challenge is to identify potential factors causing undesired inappropriate or prolonged (e.g. pathological) anxiety [132] and to take measure to reduce or remove them.

The principle of the 5 freedoms has recently been criticized by Korte and colleagues [133], who direct attention to allostasis, i.e. the capacity of the animal to change. In this concept, the animals' welfare is not at stake if they are able to meet environmental challenges, i.e. "when the regulatory range of allostatic mechanisms matches the environmental demands" [133]. Barnard introduced the concept of evolutionary salient welfare, in which welfare is defined as adaptive self expenditure, i.e. the ability of an animal to conduct itself in concordance with its adaptive life history strategy. Welfare in this view is at stake if the animal cannot fulfil its adaptive needs and is deterred from making its own decisions [69]. However, irrespective of which theoretical framework is favored, criteria of animal welfare based on sound scientific evidence are urgently needed to guide the researcher's estimate of suffering involved in animal experimentation.

#### Regulations and guidelines

In the evaluation process of models for neurobehavioral disorders, special attention should be given to detecting, and wherever possible minimizing pain, suffering, distress, sensorimotor disability and anxiety. Although most people share similar ethical values, they can be specified in different ways [134], and one may wonder how much consensus can be reached concerning ethical criteria for evaluating animal models [135]. Regulations have been established and guiding questionnaires have been developed regarding the ethics of animal studies (e.g., European Union's Directive 86/609/EEC on the Protection of Animals used for Experimental and other Scientific Purposes; USA: Animal Welfare Act, [136]; Australia: Australian code and practice for the care and use of animals for scientific purposes, Canberra: Australian Government Publishing Service, 1990). Moreover, an evaluation system proposed by Stafleu and colleagues [137] may help to decide on the ethical acceptability of intended animal experimentation. This evaluation system takes the aim and relevance of a study, human interests and the degree of potential discomfort and harm of the animals into consideration. Similarly, Broom and Johnson [138] listed measures indicating good and poor welfare that were used by Scharmann [139] for the development of humane endpoints in animal models.

At least a "silent" consensus exists in countries implementing the above mentioned regulations and guidelines that a minimum welfare of the animals being used, the benefits for animals and humans, the statistical power of the experimental approaches, and the availability of alternative in vitro or in silico (e.g. computer simulations) methods must be considered (Ethical guidelines of the international, professional society devoted to the scientific study of applied animal behaviour ISAE [140]). Most, if not all researchers involved in animal research will strive to perform good science in accordance with ethical criteria. Their own ethical values and definitions of humane endpoints will, however, set the limits of what consequences of experimental manipulations are judged as acceptable against the intended goals and expected gain of knowledge [117]. In other words, benefits must outweigh the ethical costs of the animals. These costs include pain and suffering, distress and death.

**Figure 4:**
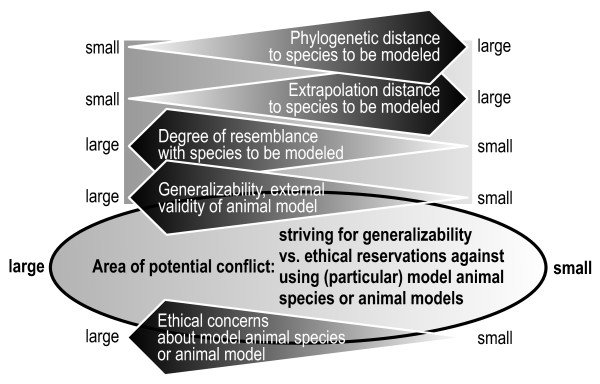
**Area of potential conflict between the choice of a specific model animal species/animal model and the expected degree of generalizability of the results obtained and the ethical reservations against using a particular model animal species/animal model**.

A formal ethical evaluation usually is performed by an independent ethics committee based on a protocol of the intended study and a thorough estimate of the adequacy of the projected animal model, the intended experimental manipulations, and in particular the choice of the model animal species [141]. It is a difficult endeavour to extrapolate results, obtained using a simple system, to a more complex system. The larger the distance between the model animal and the species to be modelled (the extrapolation distance), the poorer the generalizability of a study may be. However, a small phylogenetic or extrapolation distance *per se *does not guarantee generalizability [7,142]. The choice of a model species and ethical reservations against using the model species are an area of potential conflict (see Fig. 4).

#### Welfare concerns with respect to genetically modified animals

Adherence to the principles of the 3 Rs (refinement, reduction and replacement) is commonly accepted as an ethical guideline in the conception and execution of animal experimental studies. The principles of the 3 Rs are an attempt to promote and improve humanity in experiments involving animals, and to increase the validity of experimental results [116]. The implementation of one of the principles may, however, conflict with (one of) the other two when putting the principles into practice. This might e.g. be the case when developing models based on genetically modified animals (see [117]). This strategy is considered as a refinement (i.e. certain aspects of a disease may be mimicked more closely in these animals than in animals that had undergone other experimental manipulations), but might counteract the principle of reduction (see [117]). Large numbers of animals may be needed to establish and maintain a genetically modified line. Many of the animals required are surplus animals that will never be tested.

Moreover, the insertion or deletion of genes may interfere with normal functioning in an unexpected way [117,121,143]. Discomfort may interfere with the assessment of experimentally induced specific dysfunctions, in particular, if these dysfunctions are subtle [30]. Genetic animal models of neurobehavioral disorders that are based on conditional gene targeting techniques may not only improve the specificity and validity through their improved temporal and spatial control of the gene recombination [144], but they may also contribute to reducing discomfort.

#### Housing of animals

Housing animals in an enriched environment is one of the measures believed to improve animal welfare [145]. In a number of mouse studies it has been shown that environmental enrichment did not increase the variability and did not compromise the reliability of results (e.g. [146-148]). The authors conclude that there are no reasons why model animals should not be kept in enriched environments as standard housing condition. However, it cannot be taken for granted that different environmental conditions will not affect the expression of (endo)phenotypes differently (see for example [149,150]). This may even apply to subtle variations of the cage environment [151]. Consequently, the role of the environment (including the testing environment) must be addressed empirically as part of the validation process of animal models (in systematic or differential replication studies; see Fig. 2); investigation of the gene-environment interaction is crucial for detecting the environmental triggers for these interactions [152] and for understanding their relevance for the expression of a behavioural trait.

Within this context, the recent development of automated "phenotyping" systems and enriched housing is of interest. In an attempt to decrease the experimenter bias (observer-dependent variability), automated home cage based "phenotyping" systems (e.g. [153-157]) are being developed that allow collecting data over a long period of time simultaneously in many animals, without disturbance or interference by a human observer. While these systems may help to increase comparability between studies both within and between laboratories, they will not replace the human observer, owing to the fact that they rely on a restricted set of observational categories, and that they cannot judge whether animal welfare is at stake. A close health and welfare monitoring routine by an experienced stockman or veterinarian that parallels the automatic registrations is therefore mandatory.

#### Power of the experimental approach

Another important aspect in the ethical evaluation of animal models concerns the number of individuals needed for sound statistical analyses (e.g. [158-161]). An estimate can be achieved by applying appropriate power-analysis [162,163]. Unfortunately, sometimes the number of available animals is restricted (e.g. due to poor breeding success) and individual studies might therefore be underpowered. In that case, successful replication studies can help to increase the confidence in the results obtained in small studies (see also "Replications in model building and model validation").

Sustained awareness concerning animal welfare will sharpen the attention of the researcher to detect compromised welfare. Each ethical evaluation must include scientific reasoning (e.g. [164]). In the evaluation of animal models, assessment of the research hypothesis and the experimental design is necessary since scientifically non-valid approaches are unethical (e.g. [135]). Ethical considerations should constitute a major element from the first stage of the model building process onward. It is the obligation of everyone involved in animal experimental studies to assure the lowest possible impact of experimental manipulations on animal welfare.

### Iterative model building

**Figure 5:**
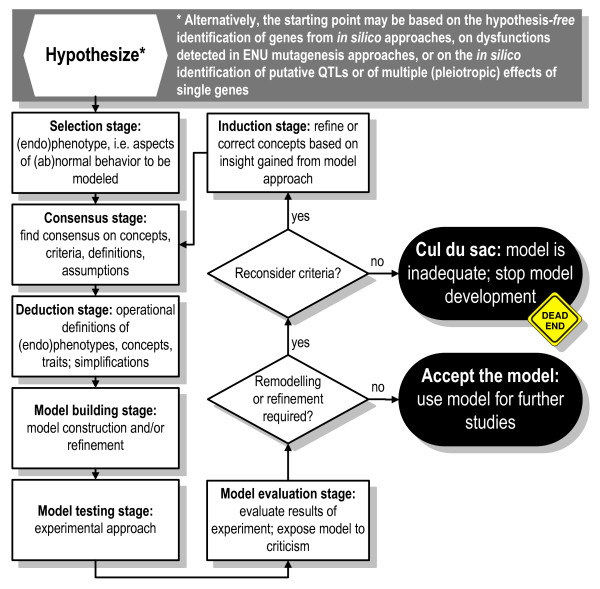
**Flow diagram depicting model building as an iterative process (inspired by **[165]; **modified after: **[10]**)**. The model evaluation stage is further elaborated in Fig. 6.

Model building can be considered as an iterative process [10,51,165] (see Fig. 5). One can perceive abduction, deduction and induction as the three elementary kinds of reasoning steps in the formulation and testing of scientific hypotheses or theories. Abduction is the process of forming new ideas and explanatory hypotheses, based on evaluating a large base of facts; it can be considered as the path from facts to theory, as a process of discovery. During the process of deduction, hypotheses based on the theory become more focused. Induction is the experimental evaluation of the hypotheses; it can be considered as path from theory to facts that ideally confirm the theory [166]. It is obvious that these steps are not independent from one another; theories evolve from observations and are supported by experimental data from experiments designed for testing hypotheses. Models are deductive and inductive tools that advance knowledge. Insight gained from a relevant animal model may affect the perception of disease symptoms and their underlying courses and processes in patients. This in turn may prompt preclinical investigators to revise, refine or extend their animal model [60] in the iterative process of model building.

The starting point of model building may be a hypothesis that has been derived via induction, i.e. the reasoning from data to ideas (e.g. psychiatric and neurological nosology, therapeutic criteria, identified endophenotypes; (see [17,167]), or abduction or deduction, i.e. the reasoning from ideas to data (e.g. observed behavioral abnormalities; induced or naturally occurring mutations [168]). The first phase of phenotyping of the animal model should be complemented with systematic observations (and eventually, specific tests for detecting sensorimotor dysfunctions) that allow monitoring and assessing the welfare of the model animal [23,121]; see below).

The quality and interpretability of the hierarchy of tests used to detect and characterize the phenotype is of crucial relevance for the next steps in the model development. In particular, data from a screen should already allow the formulation of specific hypotheses. Alternatively, the *hypothesis-free *identification of genes from *in silico *approaches (e.g. [169]), or the detection of aberrant (behavioral) phenotypes in systematic screens (e.g. ENU-mutagenesis approaches: [96,170-178]) and confirmation of these phenotypes as inherited may serve as starting point of model building. Moreover, many websites provide links to a large number of relevant genotyping and phenotyping databases (e.g. [179-181]) that may serve as starting point for identifying putative animal models. Recent bio- and neuroinformatics approaches allow the *in silico *identification of QTLs and multiple (pleiotropic) effects of single genes, without any *a priori *hypothesis [182]. *In vivo *verification of the function of the identified gene(s) and their hypothesized functions is required [183].

Irrespective of the starting point chosen for model building, it must become hypothesis driven to yield meaningful and interpretable data [184]. As Massoud and colleagues correctly state, "A model is an *invention*, not a *discovery*" ([26], p. 277), and consequently, its validity and relevance need scientific proof. The different stages of model building are the selection stage, consensus stage, deduction stage, model building stage, model testing stage, the model evaluation stage, and the induction stage. These stages have been elaborated and explained in [10] and are depicted in Fig. 5. We shall further elaborate on the model evaluation stage and apply the proposed procedure, i.e. the workflow to evaluate models, in a worked example on the rat with neonatal hippocampal lesions as a model of schizophrenia.

### Model evaluation

**Figure 6:**
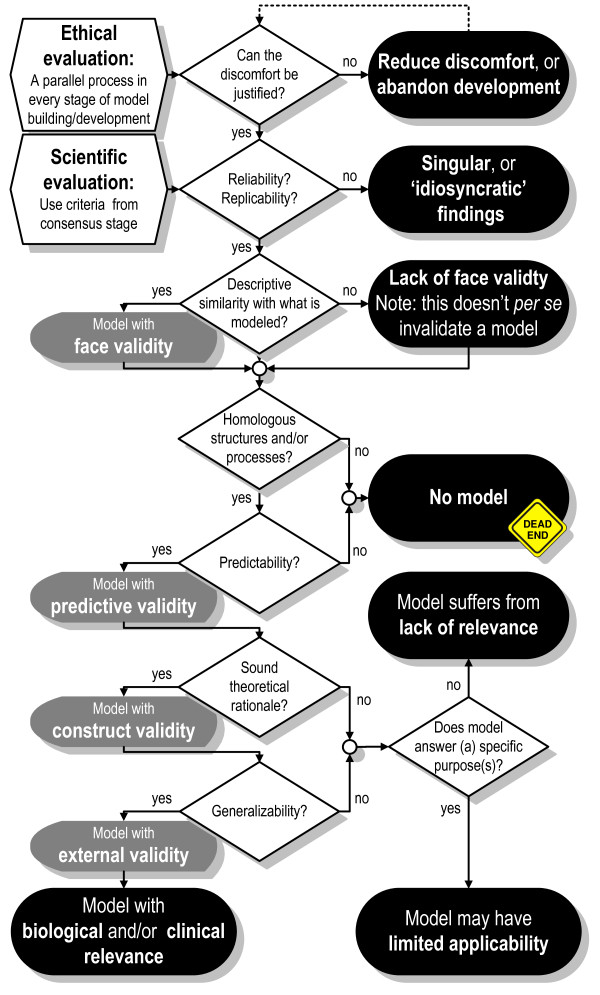
**Evaluation of an animal model using ethical and scientific evaluation criteria**.

In the *Model evaluation stage *the results obtained in the testing stage are critically discussed and evaluated. A proposed *modus operandi *for evaluating an animal model is elaborated below and depicted schematically in Fig. 6. The *relevance *of the model should be a central point in the evaluation stage. Relevance of the selected model is an explicit criterion in many guidelines for animal care and use, although evaluation rules typically remain undefined. The relevance criterion should extend to animal model development and evaluation, with the constraint that in an early stage of the model development, the *anticipated *relevance serves as criterion. Making explicit the steps of model building helps to identify the weaknesses of an animal model and to address them systematically using scientific methods. However, the purpose of a model defines the criteria that an animal model must fulfill before it can be considered as valid [64]. Consequently, any scheme for model evaluation must take into account the purposes and needs that a model is supposed to fulfill, and the questions it is expected to answer in order to determine the weights assigned to the different evaluation criteria.

Preceding the evaluation process according to scientific criteria, one may ask the ethical question whether the degree of discomfort shown by the model animal as consequence of the experimental manipulations is acceptable, considering the expected gain of knowledge [117] (see Fig. 6).

The model evaluation stage continues with the question whether the data obtained in the model are reliable and replicable, i.e. deficits must be replicably inducible, and the resulting behavioral dysfunctions must be measurable using reliable methods [72,83,94]. If the criterion of replicability is not met, then findings must be considered as singular or 'idiosyncratic'. It is most appropriate to determine the replicability of results by performing a *close *replication in an early stage of the model building process.

Next, the face validity of the model is addressed. Face validity is a criterion that some researchers believe to be of major importance (e.g. [1,49]). However, it is of greater importance that the model involves structures and processes homologous to those involved in the condition being modeled. The model is judged as invalid if neither face validity nor homologous structures and processes can be demonstrated. In this case, further development of the model should be abdicated. If the putative animal model doesn't only exhibits characteristics of the neurobehavioral disorder to be modeled, but also abnormalities that are not symptomatic, then it needs to be viewed critically [185] and one may even consider discontinuation of further development.

Then, the question must be addressed whether the putative model possesses predictive validity, i.e. whether it allows predictions to be made about what it is supposed to model. Geyer and Markou [186] consider predictive validity (and its reliability) as the only necessary criterion for the initial evaluation of any animal model for use in research. A model must have predictive validity, irrespective of whether it is considered in the broad or narrow sense (the latter being the case in most psychopharmacological studies). Enabling predictions is one of the basic purposes of any animal model (see the definition). If the model in development doesn't fulfill the criterion of predictive validity, then it doesn't meet an indispensable basic condition, and consequently, one should deliberate about abandoning further expenditures.

Construct validity, the question whether the model has a sound theoretical base, is evaluated in the next step of the validation process. Models of neurobehavioral disorders must satisfy criteria developed by basic and clinical experts [112], i.e. by scientists of diverse disciplines, such as clinicians, pathologists, molecular biologists, and (animal) behavioral scientists, e.g. psychiatrists, (bio)psychologists, ethologists, or behavioral pharmacologists (see also Table 1, Part C, second and third column). The development of adequate animal models of neurobehavioral disorders, unfortunately, is hampered by incomplete knowledge about the nature of the disorders and the resultant lack of clear diagnostic criteria. Typically, diseases of the mind are diagnosed using subjective behavioral tests. Specific psychiatric disorders cannot rigorously be identified by means of these diagnostics, but can only be categorized [187]. As a consequence, the translation to testables in animal models may be flawed by gaps in our knowledge of the disorder to be modeled.

The last step of the model evaluation stage deals with the generalizability/external validity of the animal model. Here, questions are addressed such as whether the model possesses validity across different housing conditions and laboratories [84,188], across different behavioral tests that are believed to measure the same underlying traits or states [54], and finally, whether it enables insight into, and predictions about these traits or states and their underlying processes in humans and/or other species than the one studied [7,189]. The extent to which an animal model possesses construct and external validity is a measure for its biological and/or clinical relevance [2]. Generalizability/external validity contains also elements of predictability.

If the putative animal model does not meet these criteria, then it still may be used to answer specific purposes. If it does not, it suffers from a lack of relevance. For example, mice carrying a human disease mutation without developing a corresponding mouse phenotype invalidate the use of that transgenic "model" to study potential therapeutics, because there is no pathology that the therapeutics could act upon. This mouse represents a "negative model" [7]. This observation, however, may raise a new question: why does the mutation cause a disease phenotype in humans but not in mice? Answering this question may contribute to understanding the pathophysiology of the disease. Note, however, that the purpose of the model in this example has changed, and that new evaluation criteria must be established to judge its relevance.

It should be apparent that multiple iterations are eventually needed to evaluate all criteria that define a relevant animal model and that it is unlikely that all questions posed during the evaluation stage can be answered in one 'decisive' study. When starting to develop an animal model, not all information necessary to adjudicate on whether the decision criteria are fulfilled may be available. In that case, a "patchwork approach" may be necessary. Similar to a jigsaw puzzle that usually will provide a good impression of the full picture long before all pieces are in place, the iterative model building procedure will reach a stage where sufficient pieces of evidence are available to make a sound decision about the quality and relevance of a model. One may decide to stop further development of a model if severe shortcomings of a model become obvious, even if complete information about a model is not yet available.

### Model evaluation in practice: rats with neonatal hippocampal lesions as a model of schizophrenia

Despite the very large number of animal models of various diseases that are currently used for researching fundamental disease processes and in drug development, very few animal models have been systematically evaluated in depth in terms of their validity and relevance (for a recent exception, see the review by Sagvolden et al. covering the spontaneous hypertension model of attention-deficit hyperactivity disorder [190]). Reviews tend to cover a group of models for a disease rather than focusing on one model, providing good information on the breadth of the field but not the information necessary to stringently evaluate a single animal model.

The rat with neonatal hippocampal lesion (NHL) is an example that has been frequently discussed in reviews in the context of schizophrenia models (e.g. [191-193]), but for which no systematic assessment of validity has taken place. In the NHL model, the hippocampus is lesioned, normally by an injection of an excitotoxin, in rats a few days after birth. The animals are then returned to their mothers, weaned normally, and tested as adults. This procedure induces various behavioral deficits in tests used in schizophrenia models, including deficient prepulse inhibition and hyperresponsivity to amphetamine, as well as neurodevelopmental alterations, as described below. Following the flow chart seen in Fig. 6, we can make a first effort to answer some of the questions asked for this model, though the assessment below is by no means exhaustive.

Ethical considerations of animal suffering will differ between scientists and between governing bodies, but the high prevalence of schizophrenia (approximately 1% of the general population, [194]) and the debilitating effects of the disease for patients are strong arguments for the necessity of conducting animal model-based research. The NHL model involves a number of stressors: maternal separation before surgery, placement under anesthesia, surgery, postoperative recovery, and presumably postoperative discomfort and pain. Pain and stress are not explicitly part of the NHL model, and should therefore be eliminated where possible. An obvious area for reduction of animal suffering is post-operative pain relief.

The reliability and replicability of the NHL model is seen in the replication of certain deficits across multiple sites, such as prepulse inhibition deficits and hypersensitivity to dopamine agonist-induced attenuation of prepulse inhibition [189,195-198]. Clearly this "portability" of the protocol is a crucial measure of replicability. The NHL model, however, suffers from the same issue of underreporting of negative results as many other current biomedical models: it is unknown whether attempts were made to replicate the results which failed but were not published.

Face validity of a model of schizophrenia in terms of mimicking symptomatology is exceptionally difficult, as a large portion of the hallmark symptoms of schizophrenia in patients can only be ascertained by speaking with the patient or by subjective reporting by the patient, for instance hallucinations, delusions and flattened affect. It has been argued that the NHL model has face validity based on a number of characteristics seen in schizophrenics which are also seen in NHL model rats, such as deficits in prepulse inhibition and latent inhibition, and cellular, molecular and morphological changes in the brain [199]. While these alterations are indeed present in patients, they are neither exclusive to schizophrenia nor are they key symptoms of the disease. It may prove to be impossible to produce an animal model with face validity for schizophrenia, at least for the positive and negative symptoms.

The rationale for the use of the NHL model is anchored in the idea of homologous brain structures being responsible for the disease and for NHL-induced deficits, the next criterion in our evaluation. The hippocampus, which is lesioned in the NHL model, has frequently been reported to have a reduced volume in schizophrenic patients [200-205]. Alterations in volume and neurotransmitter content in the prefrontal cortex were also repeatedly found in schizophrenic patients (reviewed in [206]. Similarly, rats with NHLs show altered prefrontal cortical development, both in terms of structure [207,208], and function [207-210]. Thus the model does seem to affect key brain areas that are affected in schizophrenia.

The predictive validity for animal models of schizophrenia has also proven difficult, particularly as predictive validity is understood in psychopharmacology – that is, a model is considered predictive if it can predict which drugs will be effective in treating the disease modeled. The NHL model has been shown to be sensitive to a long list of both typical and atypical antipsychotics which are in clinical use today [197,211,212]. However, all antipsychotics currently marketed are based on the same basic pharmacological mechanisms: dopamine D_2 _receptor antagonism or partial agonism, in some cases coupled with activity at various serotonin receptors. In all animal models of schizophrenia, it is therefore difficult to conclude whether a model can predict effective treatment, or if it simply relies on the same receptor set and spuriously correlates with clinical efficacy. An intriguing recent development in schizophrenia treatment is a clinical trial showing efficacy of the metabotropic glutamate receptor 2 agonist LY404039 in symptom relief in schizophrenics [213]. LY404039 relies on a different pharmacological substrate than previous antipsychotics. It will be of interest to see whether the NHL model would predict the clinical efficacy that has been seen in clinical trials with this drug.

As mentioned in the explanation of the workflow, evaluation of construct validity of animal models of psychiatric diseases (including schizophrenia) is exceptionally difficult, as the exact, most likely multifactorial, ethiology is not known. The rooting of the NHL model in neurobiological substrates that are known to be involved in schizophrenia contributes to it construct validity. However, a major hurdle for the model is that, while it has been hypothesized that neurodevelopmental processes play an important role in schizophrenia [191], the appearance of symptomatology in late adolescence in patients precludes systematic studies of neurobiology of future schizophrenics during early development, thus we do not know if schizophrenic patients show damage or alterations in the hippocampus during this period. Furthermore, given the strong genetic link found in family members of schizophrenics [214], it is highly unlikely that a traumatic event such as lesioning is responsible for hippocampal alterations seen in later life in schizophrenic patients.

The generalizability of the NHL model appears to be good, as it transfers across laboratories and species, as well as producing effects in various tests frequently used in preclinical testing of antipsychotics. As was briefly mentioned above in the assessment of replication and reliability, effects on a number of tests have been replicated in several laboratories. Furthermore, the model produces behavioral effects in prepulse inhibition, hyperreactivity to dopamine agonists, and deficits in latent inhibition [191], all of which are frequently used tests for assessing antipsychotic activity. Finally, the model does seem to generalize to non-human primates, where neonatal hippocampal lesions produce deficits in adult animals similar to those seen in NHL rats (reviewed in [215]).

To place the above initial evaluation of the NHL model in the framework of the workflow proposed in Fig. 4, we arrive at the following:

● The discomfort produced by the model can be ethically justified, though proper precautions to minimize discomfort must be taken.

● The model has been replicated and reproduced at multiple locations.

● The face validity for key symptoms of schizophrenia is lacking, because of the inherent inability for modeling these symptoms in animals.

● Brain structures damaged in the model, either by lesion or by resultant developmental abnormalities, are homologous to areas which also show abnormalities in schizophrenia.

● The predictive validity of the model viewed in terms of predicting drug efficacy is good for the classes which are already in clinical use, but the model will need to prove itself by predicting novel drug classes. This may, in fact, be expected from a model with good construct validity [21].

● The theoretical rationale (construct validity), however, is unsatisfactory, as the lesioning method is likely to induce structural/functional abnormalities of the hippocampus and its projections areas, but it most likely doesn't mimic developmental abnormalities (which are as yet not understood) in children who will later develop schizophrenia.

● The generalizability (external validity) of the model across different laboratories, tests, and species is well established, at least with respect to the classes of prescribed antipsychotics.

Following the workflow, this leads us to the question "does the model answer a specific purpose"? The NHL model is one of several models currently in use for behavioral pharmacological assessment of antipsychotic compounds. The problems faced by this model in terms of face validity and construct validity are likely to be faced by any behavioral pharmacological model, because we simply do not have the ability to test for key symptoms in animals, nor do we have the knowledge of the ethiology of the disease to produce an animal model that fulfills all of the criteria set forth in the workflow. Given its good predictive validity with antipsychotic compounds with proven therapeutic efficacy, the model's basis in homologous brain structures and its good generalizability, the model can be used for the specific purpose of testing compounds for their potential to alleviate symptoms of schizophrenia. However, the unascertained construct validity means that care should be taken in using the model to uncover fundamental disease processes and novel therapeutic approaches.

#### "Standard models"

Some of the (transgenic) animal models have gained the status of "standard" animal model for a particular disease. Recently, the transgenic SOD1G93A mice, considered as "standard" mouse model for amyotrophic lateral sclerosis (ALS), a paralytic neurodegenerative disorder in humans (see [216,217]) has been up to debate. Doubts arose about the relevance of this model for identifying putative therapeutics for the treatment of ALS (commented by [218]). The model is based 1) on a point mutation of the human superoxide dismutase (SOD1) gene in the familial ALS form, and 2) on experimental evidence that a number of putative therapeutics appear to be able to prolong survival in transgenic mice carrying 23 copies of this human gene mutation. However, to date, putative therapeutics that were effective in this animal model have been ineffective in clinical trials in ALS patients, mooting the value of the SOD1 mouse for identifying therapeutics for familial and sporadic ALS. One may conclude that the relevance of this animal model still needs to be shown [216], as neither the criterion of predictive validity nor the criterion of generalizability of results has been met (e.g. does an animal of the familial form of ALS generalize to the sporadic forms of the disease?). This example illustrates the need for extended, multi-tiered and systematic validation of animal models.

Similar doubts recently arose concerning the value of animal models in stroke research (e.g. [76,219]), mainly because the majority of compounds with confirmed neuroprotective efficacy in these models appeared to be ineffective in human clinical trials. One of the factors might be that the standard animal models, such as rodents with focal or global ischemia induced by occlusions of brain arteries do not mimic the pathology in humans with sufficient fidelity, i.e. that they suffer from poor construct validity.

If the scientific criteria of the model are not fulfilled, then animals may still be used for *in vivo *screening of putative therapeutics, based on the observation that correlated responses have been found in animals and humans [27]. Such correlations can generate theories about the underlying mechanisms of action and hence testable hypotheses. Willner [49,220] contrasted the animal model with two other, closely related, experimental methodologies. The first one was *drug screening*, and the second was *behavioral bioassay*. Drug screening tests are designed to distinguish between potentially effective and ineffective drugs (e.g. [221,222]) whereas behavioral bioassays are designed to assess the functional state of, for example, a specific brain system, or to explore the neurobiological specificity of compounds and their molecular and cellular mechanism of action (e.g. in drug discrimination paradigms [223]; see also Table 1, Part A, first column). Drug screening and behavioral bioassay are two experimental methodologies, distinct from animal models, but they are not mutually exclusive. There is a fluent transition from drug screening and behavioral bioassay to animal models: the more precise the assumptions (and/or the knowledge) about underlying relations and processes, the more the criteria for an animal model may be fulfilled.

## Conclusion

Unfortunately, no consensus exists about the order and weight of the different steps that are necessary for developing an animal model, nor are there common, generally accepted criteria for evaluating the resulting putative model. Perceiving model building as an iterative multi-stage process with an evaluation stage with predefined appraisal criteria may guide the scientists through the model building and model evaluation process. The suggested workflow can also be used to develop and/or evaluate animal models in other areas of research. In almost the same manner as animal models can be improved, guided by the procedure outlined above, the developmental and evaluation procedure itself may be improved by careful definition of the purpose(s) of a model and by defining better evaluation criteria.

## Competing interests

The authors declare that they have no competing interests.

## Authors' contributions

FJS conceived the review, SSA elaborated the ethical aspects of model building and model evaluation, and REN evaluated the neonatal hippocampal lesion model of schizophrenia along the model evaluation procedure expanded in this article. All authors have read and approved the final manuscript.
